# 基于深度学习的人工智能胸部CT肺结节检测效能评估

**DOI:** 10.3779/j.issn.1009-3419.2019.06.02

**Published:** 2019-06-20

**Authors:** 欣菱 李, 芳芳 郭, 振 周, 番栋 张, 卿 王, 志君 彭, 大同 苏, 亚光 范, 颖 王

**Affiliations:** 1 300052 天津，天津医科大学总医院放射科 Department of Radiology, Tianjin Medical University General Hospital, Tianjin 300052, China; 2 453100 新乡，新乡医学院第一附属医院放射科 Department of Radiology, the First Affiliated Hospital of XinXiang Medical College, Xinxiang 453100, China; 3 100080 北京，Deepwise Healthcare Deepwise Healthcare, Beijing 100080, China; 4 300052 天津，天津医科大学总医院，天津市肺癌研究所，天津市肺癌转移与肿瘤微环境重点实验室 Tianjin Key Laboratory of Lung Cancer Metastasis and Tumor Microenvironment, Tianjin Lung Cancer Institute, Tianjin Medical University General Hospital, Tianjin 300052, China

**Keywords:** 计算机体层成像, 肺结节, 深度学习, 人工智能, 检出, Computed tomography, Lung nodules, Deep learning, Artificial intelligence, Detection

## Abstract

**背景与目的:**

肺结节精确检测是实现肺癌早诊的基础。基于深度学习的人工智能在肺内结节检测领域发展迅速，对其效能进行验证是促进其应用于临床的前提。本研究旨在评估基于深度学习技术的人工智能软件在胸部计算机断层扫描（computed tomography, CT）恶性及非钙化结节检出中的价值。

**方法:**

由天津医科大学总医院自建胸部CT肺结节数据库中随机抽取200例胸部CT数据，包含病理证实的肺癌及随访结节病例，导入肺结节人工智能识别系统，记录软件自动识别结节，并与原始影像报告结果进行对比。人工智能软件及阅片者检测到的结节由2名胸部专家进行评估并记录其大小及特征。计算灵敏度、假阳性率评估人工智能软件及医师的结节检测效能，应用*McNemar*检验确定二者之间是否存在显著性差异。

**结果:**

200例胸部多层螺旋CT共包含非钙化结节889枚，其中肺癌结节133枚，小于5 mm结节442枚。人工智能及放射科医师肺癌检出率皆为100%。人工智能软件结节检测灵敏度明显高于放射科医师（99.1% *vs* 43%, *P* < 0.001）。人工智能总体假阳性率为每例CT 4.9个，排除5 mm以下结节后降为1.5个。

**结论:**

基于深度学习的人工智能软件能实现恶性肺结节的无漏诊检出，具有较医师更高的结节检出灵敏度，在排除微小结节后可降低假阳性率。

## Abstract (en)

**Background and objective:**

The detection of pulmonary nodules is a key step to achieving the early diagnosis and therapy of lung cancer. Deep learning based Artificial intelligence (AI) presents as the state of the art in the area of nodule detection, however, a validation with clinical data is necessary for further application. Therefore, the aim of this study is to evaluate the performance of AI in the detection of malignant and non-calcified nodules in chest CT.

**Methods:**

Two hundred chest computed tomography (CT) data were randomly selected from a self-built nodule database from Tianjin Medical University General Hospital. Both the pathology confirmed lung cancers and the nodules in the process of follow-up were included. All CTs were processed by AI and the results were compared with that of radiologists retrieved from the original medical reports. The ground truths were further determined by two experienced radiologists. The size and characteristics of the nodules were evaluated as well. The sensitivity and false positive rate were used to evaluate the effectiveness of AI and radiologists in detecting nodules. The McNemar test was used to determine whether there was a significant difference.

**Results:**

A total of 889 non-calcified nodules were determined by experts on chest CT, including 133 lung cancers. Of them, 442 nodules were less than 5 mm. The cancer detection rates of AI and radiologists are 100%. The sensitivity of AI on nodule detection was significantly higher than that of radiologists (99.1% *vs* 43%, *P* < 0.001). The false-positive rate of AI was 4.9 per CT and decreased to 1.5 when nodules less than 5 mm were excluded.

**Conclusion:**

AI achieves the detection of all malignancies and improve the sensitivity of pulmonary nodules detection beyond radiologists, with a low false positive rate after excluding small nodules.

肺癌是对人类健康和生命威胁最大的恶性肿瘤之一^[1]^。降低肺癌死亡率、提高生存质量的关键是早诊早治。肺结节是肺癌的早期表现，其检出率随着薄层计算机断层扫描（computed tomography, CT）技术的发展而提高，但明显增多的CT数据也增加了医师的阅片负担，从而可能导致肺结节的漏诊^[2]^。应用人工智能（artificial intelligence, AI）技术对海量CT图像进行初步筛查并标记可疑病变，可以帮助医师减少工作量并提高诊断准确率^[3, 4]^。

本研究旨在评估基于深度学习的人工智能软件在胸部CT恶性及非钙化结节检测中的应用价值。

## 1 资料与方法

### 1.1 研究对象

自天津医科大学总医院自建肺结节病例库中随机抽取200例CT数据，包含有病理结果或随访两年以上无变化的结节病例。图像纳入标准：①至少包含1枚非钙化结节；②有薄层胸部CT图像（层厚≤1.25 mm）；③排除患有弥漫性转移、间质性肺病，广泛瘢痕形成、肺炎、纤维化、肺水肿和具有严重运动伪影的图像。

### 1.2 CT扫描技术

所有检查由16排（General Electric Company, GE）或64排（Light Speed VCT, Discovery HDCT, Optima）螺旋CT机进行扫描。扫描范围自胸廓入口至肺底部，患者一次吸气后屏气完成全肺扫描。扫描方式：螺旋扫描；管电压120 kV或140 kV；管电流：200 mA-340 mA；螺距：1.375:1；层厚：5.0 mm；图像矩阵：512×512；视野（field of view, FOV）：360 mm。使用标准算法重建1.25 mm层厚轴位图像。

### 1.3 肺结节检测

基于深度学习模型的人工智能软件（Deepwise healthcare, V190120）由深睿医疗公司提供，将200例胸部CT原始数据传输至工作站，软件系统自动批量进行肺结节识别和标记。影像医师的结节检测基于存档的影像报告，报告由高年资医师对低年资医师的初始报告进行审核后完成。

### 1.4 结节标准认定

由2名胸部影像专家在参考AI及影像报告结果的前提下，在横断面进行观察，确定可疑病灶位置后，参考多平面重建、最大密度投影等结果来进一步确定标注结果与肺内结节的定义是否接近，从而做出判断，以2人的一致性意见作为真结节金标准，同时记录每个非钙化结节的大小、位置和密度。结节的大小分为3组： < 5 mm、5 mm-10 mm和 > 10 mm。结节所在位置分成四组：与胸膜相连、周围性结节（距胸膜2 cm以内，但不与胸膜相连）、肺门区结节（距肺门2 cm以内）和中心性结节（位置介于周围性和肺门区结节之间）。根据结节密度分为三组：实性结节、部分实性结节和磨玻璃密度结节。磨玻璃密度结节被定义为肺内模糊的、密度稍大于肺组织而未掩盖支气管、血管等肺纹理结构的结节；部分实性结节被定义为结节内既包含磨玻璃密度成分，同时也包含实性软组织密度成分^[5, 6]^。

### 1.5 统计学方法

应用SPSS 17.0统计学软件进行资料录入、整理及统计学分析。分别计算AI和影像医师检出非钙化肺结节的灵敏度及假阳性率。基于 < 5 mm结节临床意义很小，假阳性率统计时分别计算总体及将 < 5 mm结节排除后的数值。应用*McNemar*检验比较深度学习模型、影像医师检出肺结节的能力，*P* < 0.05为差异有统计学意义。

## 2 结果

### 2.1 结节大小及特征

入选200例CT图像经两位影像专家确认共包含889个真结节，其中恶性结节133例，均经病理证实。结节大小及特征见表 1。

**1:** 889枚真结节大小及特征的检出情况 Characteristics of 889 true pulmonary nodules

Variable	*n*	Nodules detected by		*P*
		AI (%)	Radiologists (%)	
Size				
< 5 mm	412	404 (98.05)	66 (16.02)	0.000
5 mm-10 mm	283	283 (100.00)	133 (47.00)	0.000
> 10 mm	194	194 (100.00)	186 (95.88)	0.008
Density				
Soild	498	491 (98.59)	188 (37.75)	0.000
Part-soild	81	81 (100.00)	80 (98.76)	1.000
Ground-glass	310	309 (99.68)	127 (40.96)	0.000
Location				
Subpleural	168	164 (97.62)	84 (50.00)	0.000
Peripheral area	514	511 (99.42)	217 (42.22)	0.000
Intermediate area	179	178 (99.44)	75 (41.90)	0.000
Hilar area	28	28 (100.00)	9 (32.14)	0.000
AI: artificial intelligence.				

### 2.2 基于深度学习的人工智能软件与影像医师结节检出灵敏度

对于133例恶性结节，AI与影像医师的检出率均为100%。AI总体肺结节检测灵敏度明显高于影像医师（99.1% *vs* 43%, *χ*^2^=483.20, *P* < 0.001）（表 1）。相对于医师，AI额外检测出的结节多为直径 < 5 mm及5 mm-10 mm的结节、实性及磨玻璃密度结节。对于亚实性结节（图 1），AI的检出率稍高，但出率没有显著性差异。不同位置的结节，AI的检出率均高于影像医师（表 1）。AI共漏检了8例结节，均为 < 5 mm结节。

**1:**
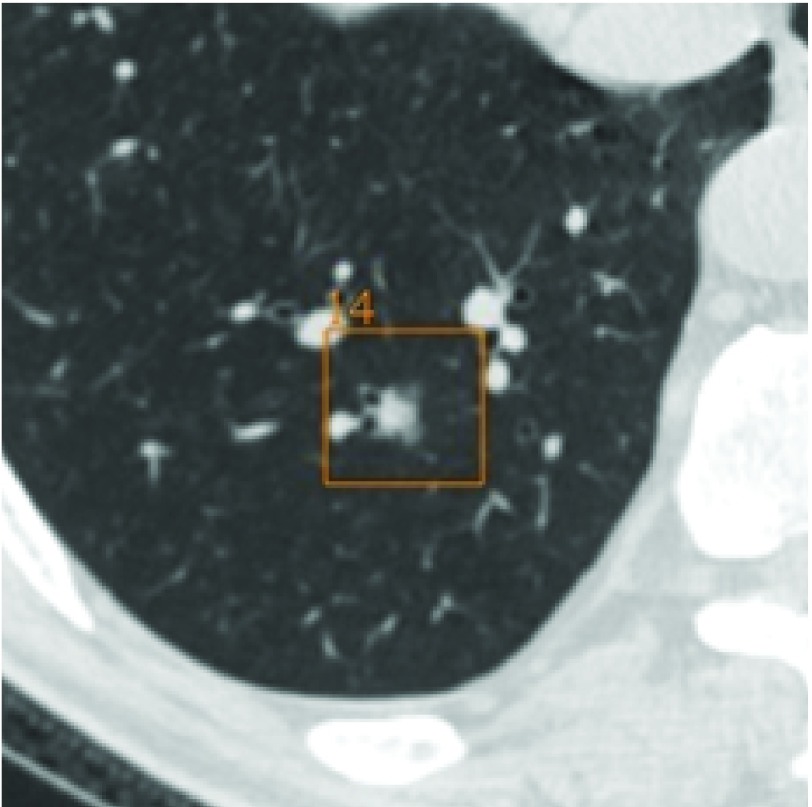
右肺下叶部分实性结节，与细支气管相连，直径10.7 mm，距胸膜43.3 mm，影像医师漏诊，AI实现检测。 A part-solid nodule located in right lower lobe, connected to the bronchioles, 10.7 mm in diameter, 43.3 mm far from the pleura, missed by radiologist, but detected by AI.

### 2.3 基于深度学习的人工智能软件与影像医师结节检测的假阳性率

深度学习模型的假阳性结节数为993个，假阳性率为每例CT 4.9个结节。若排除直径 < 5 mm的假阳性结节，其假阳性率降低为每例CT 1.5个。假阳性结节中，335例（34%）表现为小叶中心性结构（图 2），295例（30%）表现为增粗、相连，聚集、弯曲、交叉及稍膨隆的血管结构，126例（13%）表现为为稍扩张、增厚的细支气管（图 3）或细支气管内分泌物，780例（8%）为片状实变或磨玻璃密度影，包括局部实变、肺血分布不均、局部肺膨胀不全及慢性炎症等，73例（7%）与索条、瘢痕等陈旧性病灶相关，30例（3%）为其他原因，如与骨性结构相关，表现为局部凸显肺野的骨性结构，局限性气体潴留，胸膜斑块等，22例（2%）与小树芽等感染性病灶病灶相关，22例（2%）与增厚的小叶间隔及小叶内间隔相关，余12例（1%）无特异性。影像医师的假阳性总计为3个结节，皆与血管结构相关，假阳性率为每例CT 0.015个。

**2:**
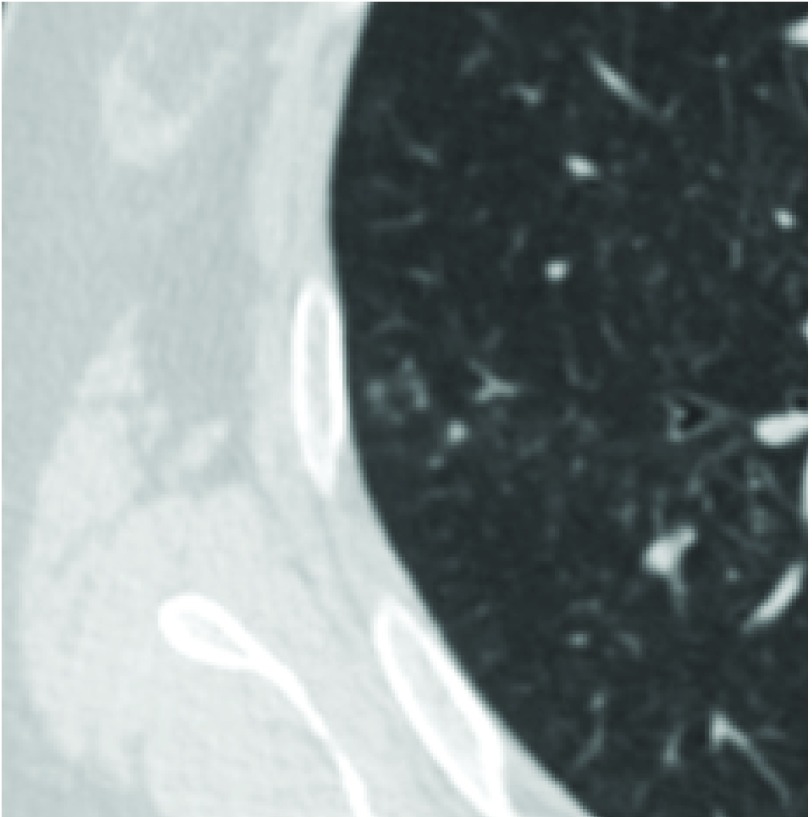
右肺上叶花环状小叶核心结构，AI假阳性。 Centrilobular nodules with a garland shape located in right upper lobe, false positive case by AI.

**3:**
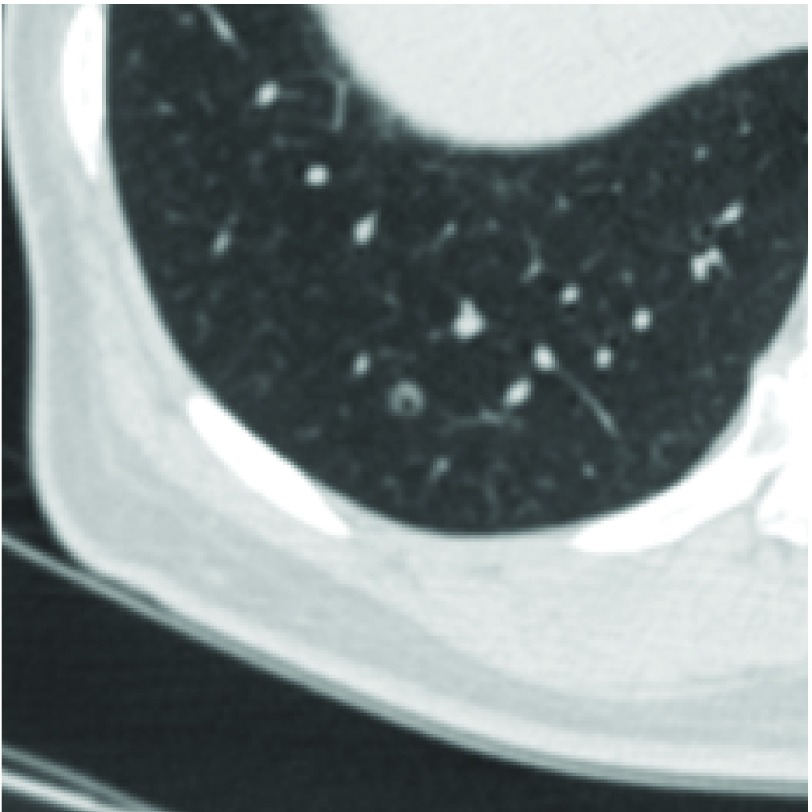
右肺下叶增厚、扩张的细支气管，AI假阳性。 Thickened, dilated bronchioles located in right lower lobe, false positive case by AI.

## 3 讨论

我们的研究结果显示AI在我们的数据集中实现肺癌的100%检测，相对于放射科医生提高了结节检测的灵敏度。AI的总体假阳性率较高，但在排除5 mm结节后，假阳性率降低到可接受的水平。

本研究中，AI及影像医师对数据集中133个恶性结节的灵敏度均为100%，说明AI及医师对相对较大的和（或）具有一定恶性可能的结节检出情况理想，体现在二者对于大于10 mm的结节及部分实性结节均具有很高的检出率（表 1）。对于所有非钙化结节的整体检出情况，AI的灵敏度较医师明显增高，尤其体现在 < 5 mm、实性结节。影像医师在灵敏度方面明显低于AI，但漏诊结节大部分为 < 5 mm的结节，其原因首先在于阅片时采用仅为水平轴位图像，小结节与血管断面轴位投影相似，容易漏诊，其次可能在于影像医师阅片时间有限或注意力无法保持全程集中，满足于较大病变的检测，对于小的病变认为临床意义极小而没有引起重视或依靠自己的经验进行了排除。AI在结节检测方面确实能够对影像医师提供帮助，但从临床应用的角度考虑，这类结节的临床意义尚有待商榷，完全按照AI的结果对此类结节进行进一步临床干预会增加医疗负担并占用医疗资源，因此，对这些结节进行进一步分析，从而判断是否需要临床处理是医生和AI需要进一步面对的问题。

我们的研究发现AI的总体结节检测假阳性率较高，尤其易误诊于小叶核心结构，其它主要误诊原因包括：与气管相关，包括增厚、扩张的细支气管，气管及细支气管内分泌物等；与血管相关，包括增粗、迂曲、交叉的血管、肺门增粗的血管等；正常或异常的肺结构如小叶核心结构，增厚的小叶间隔及小叶内间隔；各时期的感染性病灶如索条、瘢痕、树芽、片状实变及磨玻璃密度影；其他原因，如局部凸向肺野的骨性结构、局限性气体潴留、胸膜斑块等。造成假阳性的原因可能与AI设定的检出结节大小的阈值极低有关。在我们去除 < 5 mm的假阳性结节后，假阳性率减少约2/3。本研究中影像医师的假阳性率极低，其根本原因在于影像医师关于假阳性结节的判断经验明显较AI丰富，而现阶段AI的重点更集中在阳性结节的学习，对于假阳性结节的学习尚有欠缺。

AI作为当前科学技术发展中的一门广泛应用于医学领域的前沿学科，对肺结节进行检测是人工智能医疗发展的方向^[7, 8]^。深度学习是通过构建深层网络结构进行多层次特征学习的人工智能方法，对比传统的计算机辅助检测系统（computer-aided detection, CAD）系统具有优势。本研究所采用的软件基于三维卷积神经网络（3D-convolutional neural network, 3D-CNN）利用深度学习进行目标检测，能够充分利用肺结节的空间三维信息^[9]^。深度学习模型对肺门区、胸膜下区及非实性结节的检出能力明显提高^[10, 11]^，对实性结节的敏感度比文献报道中更高^[12, 13]^。深度学习模型有较快的运算速度，随着经验的不断积累、模型的不断迭代，其诊断敏感度及准确性也会不断上升，而且假阳性也会得到控制。

本研究的局限性在于数据集病例数选择标准基于回顾性的病例选取，病例均为医师发现的病例，临床中漏诊的病例无法通过回顾性病例选择获得，因此存在一定的选择性偏倚。

综上所述，基于深度学习的肺结节AI检测软件具有比医师更高的灵敏度，可作为医师的辅助检测工具进行肺结节的筛查，排除 < 5 mm结节会降低AI的假阳性率。

## References

[1] Torre LA, Bray F, Siegel RL (2015). Global cancer statistics, 2012. CA Cancer J Clin.

[2] Sui Y, Wei Y, Zhao D (2015). Computer-aided lung nodule recognition by SVM classifier based on combination of random undersampling and SMOTE. Comput Math Methods Med.

[3] Sahiner B, Chan HP, Hadjiiski LM (2009). Effect of CAD on radiologists' detection of lung nodules on thoracic CT scans: analysis of an observer performance study by nodule size. Acad Radiol.

[4] Xia Y, Lu S, Wen L (2014). Automated identification of dementia using FDG-PET imaging. Biomed Res Int.

[5] Tsutani Y, Miyata Y, Nakayama H (2012). Prognostic significance of using solid versus whole tumor size on high-resolution computed tomography for predicting pathologic malignant grade of tumors in clinical stage IA lung adenocarcinoma: a multicenter study. J Thorac Cardiovasc Surg.

[6] Lee HJ, Goo JM, Lee CH (2007). Nodular ground-glass opacities on thin-section CT: size change during follow-up and pathological results. Korean J Radiol.

[7] Setio AA, Ciompi F, Litjens G (2016). Pulmonary nodule detection in CT images: False positive reduction using multi-view convolutional networks. IEEE Trans Med Imaging.

[8] Setio A, Traverso A, de Bel T (2017). Validation, comparison, and combination of algorithms for automatic detection of pulmonary nodules in computed tomography images: The LUNA16 challenge. Med Image Anal.

[9] Kang G, Liu K, Hou B (2017). 3D multi-view convolutional neural networks for lung nodule classification. PLoS One.

[10] Beigelman-Aubry C, Hill C, Boulanger X (2009). Valuation of a computer aided detection system for lung nodules with ground glass opacity component on multidetector-row CT. J Radiol.

[11] Yanagawa M, Honda O, Yoshida S (2009). Commercially available computer-aided detection system for pulmonary nodules on thin-section images using 64 detectors-row CT: preliminary study of 48 cases. Acad Radiol.

[12] Beigelman-Aubry C, Raffy P, Yang W (2007). Computer-aided detection of solid lung nodules on follow-up MDCT screening: evaluation of detection, tracking, and reading time. AJR Am J Roentgenol.

[13] Das M, Muhlenbruch G, Heinen S (2008). Performance evaluation of a computer-aided detection algorithm for solid pulmonary nodules in low-dose and standard-dose MDCT chest examinations and its influence on radiologists. Br J Radiol.

[14] de Hoop B, van Ginneken B, Gietema H (2012). Pulmonary perifissural nodules on CT scans: rapid growth is not a predictor of malignancy. Radiology.

